# *Gondysia* preceeded *Neadysgonia* (Lepidoptera, Erebidae, Erebinae), a new generic synonymy from Southeastern United States – Corrigendum

**DOI:** 10.3897/zookeys.264.4378

**Published:** 2013-02-06

**Authors:** J. Bolling Sullivan, Albert Legrain

**Affiliations:** 1 200 Craven St., Beaufort, North Carolina 28516 USA; 2 Quai du Halage, 10, 4681-Hermalle (Liège), Belgium

In the paper cited, a list of taxa was included to summarize the taxonomic changes resulting in the synonymy of *Neadysgonia* Sullivan, 2010, with *Gondysia* Berio, 1965. A formatting change in the taxonomic changes on the list in page proof stage resulting in all the changes being shown as new synonymies instead of both new synonymies. The corrected list is given below:

*Gondysia* Berio, 1955

*Parallelia*, Auct. *nec* Hb., 1818

*Neadysgonia* Sullivan, 2010, **syn. n.**

*consobrina* (Gn., 1852), **comb. n.**

*redditura* (Walker, 1858)

*pertorrida* Berio, 1955, **syn. n.**

*similis* (Gn., 1852), **comb. n.**

*apicalis* (Gn., 1852)

*concolor* (Grt., 1893)

*smithii* (Gn., 1852), **comb. n.**

*telma* (Sullivan, 2010), **comb. n.**
